# Stable expression and phenotypic impact of *attacin E *transgene in orchard grown apple trees over a 12 year period

**DOI:** 10.1186/1472-6750-10-41

**Published:** 2010-06-03

**Authors:** Ewa Borejsza-Wysocka, John L Norelli, Herb S Aldwinckle, Mickael Malnoy

**Affiliations:** 1 Department of Plant Pathology, Cornell University, Geneva, NY 14456, USA; 2 USDA-ARS Appalachian Fruit Research Station, Kearneysville, WV 25430, USA

## Abstract

**Background:**

Transgenic trees currently are being produced by *Agrobacterium*-mediated transformation and biolistics. The future use of transformed trees on a commercial basis depends upon thorough evaluation of the potential environmental and public health risk of the modified plants, transgene stability over a prolonged period of time and the effect of the gene on tree and fruit characteristics. We studied the stability of expression and the effect on resistance to the fire blight disease of the lytic protein gene, *attacin E*, in the apple cultivar 'Galaxy' grown in the field for 12 years.

**Results:**

Using Southern and western blot analysis, we compared transgene copy number and observed stability of expression of this gene in the leaves and fruit in several transformed lines during a 12 year period. No silenced transgenic plant was detected. Also the expression of this gene resulted in an increase in resistance to fire blight throughout 12 years of orchard trial and did not affect fruit shape, size, acidity, firmness, weight or sugar level, tree morphology, leaf shape or flower morphology or color compared to the control.

**Conclusion:**

Overall, these results suggest that transgene expression in perennial species, such as fruit trees, remains stable in time and space, over extended periods and in different organs. This report shows that it is possible to improve a desirable trait in apple, such as the resistance to a pathogen, through genetic engineering, without adverse alteration of fruit characteristics and tree shape.

## Background

Genetic transformation technology has facilitated studies of gene regulation in several plant species including trees [1,2]. Some of the most problematic barriers to genetic improvement of trees, such as their large size and long breeding cycles, can be circumvented by the application of these techniques. Because trees have a long lifespan, knowledge of the genetic regulation of mature tissues is of major importance. The successful introduction of transgenic trees depends on improving the horticultural performance of the modified plants and on the stable expression of the transgene [3]. There is no need to consider the inheritance pattern to successive generations in trees, since grafting is the normal method of propagating fruit trees and unlimited numbers of T0 transgenic lines can be selected for evaluation. The study of transgene expression is of vital importance whenever transgenic plants are produced. Transgene expression levels are influenced by many factors, in particular the site of integration of the transgene within the plant genome, gene silencing, and the promoter employed [4].

In the eighteen years since apple transformation was first reported many common scion and rootstock cultivars have been successfully transformed, yet no transgenic cultivars have progressed through to commercial production. With hindsight, it was probably optimistic to have expected the latter to occur. Most early reports on transgenic apple described 'proof of concept' experiments involving the development of regeneration and transformation protocols, and the choice of appropriate promoters and selectable markers [summarized in [5,6]]. More recently, attention has focused on functional testing of traits of scientific and potential commercial interest. For commercial application of apple transformation technologies, it is imperative that horticulturally useful transgenes be stably expressed in time and space throughout the lifetime of the plant. However, many recent studies show that transgene instability frequently occurs in transgenic plants [7-10]. Even though the mechanisms of this instability, e.g. gene silencing or loss, are not fully understood, it is generally accepted that several factors, such as methylation, copy number, genome rearrangement, insertion site in genome and homology of an endogenous gene to the transgene, are responsible for transgene expression instability [11-14]. Although there have been several reports on characterization of the integration pattern, expression and inheritance of transgenes in transgenic apple plants and their progeny [2,15,16], only a few studies have been published on the expression of transgenes during long-term evaluation in the field and the impact of the transgene on fruit characteristics.

Twenty years ago, when we started our research to improve resistance to pathogens using rDNA technology we decided to investigate the expression of antimicrobial proteins in apple as a possible means of restricting the multiplication of the pathogen in the plant after the infection. Antibacterial proteins are important components of the overall antimicrobial defense mechanisms of many groups of animals, including arthropods, amphibians, and mammals [17]. Multiple compounds, probably acting in synergy, have bactericidal action on a large range of gram negative and gram positive bacteria. Attacins are antimicrobial proteins produced by *H. cecropia *in response to bacterial infection. Six different isoforms (A-F) of attacin with a molecular weight of 20-23 kDa can be fractionated according to their isoelectric points into a basic group (A, B, C, and D) and an acidic group (E and F) [18]. Attacin F is derived by proteolysis of attacin E [19]. These peptides are active against the inner membrane, or peptidoglycan, of the periplasm, and are not normally active against *E. coli *or other gram negative bacteria. Carlsson et al. [20] suggested that attacin causes an increase in the permeability of the outer membrane. Attacin was also observed to cause irregular shaped cells, irregular patterns of cell division, and lysis, which was attributed to effects on outer membrane permeability.

Attacin E under the control of inducible (Pin2) and constitutive (CaMV35S) promoters introduced into various species significantly increased resistance to bacterial pathogen infection. In apple the increase in resistance to *Erwinia amylovora *(fire blight) was correlated with the expression level of attacin [21,22], however increases in resistance in potato and pear were not correlated with expression [23,24]. The presence of a signal peptide, which allowed the secretion of attacin into the intracellular space, increased the resistance of the transgenic apple in spite of low attacin content [21,22]. Attacin E expression is increased when this gene is under the control of AMV translational enhancer [21,22]. In this paper we report the stability of the expression of the *attacin E *gene in 12 year old trees grown in the field and the impact of this gene on the apple fruit characteristics (acidity, sugar level, color, aspect, firmness, weight).

## Results

### Flowering and fruiting of transgenic apple plants

**Figure 1:**
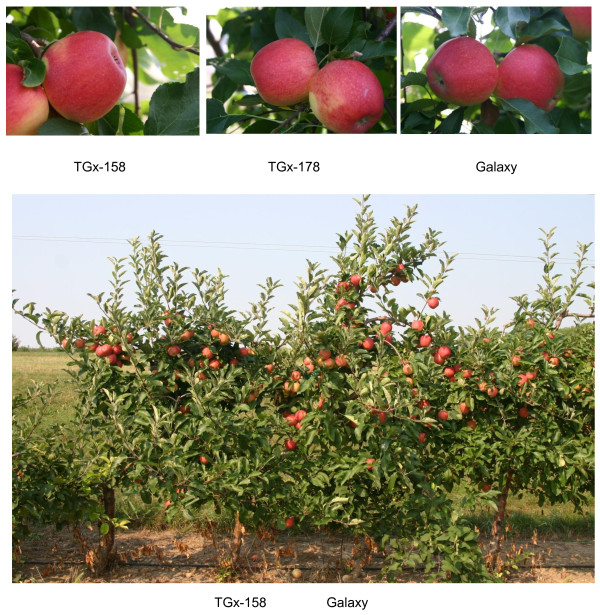
**Picture of fruits and tree from transgenic lines (TGx158 and TGx178) and control ('Galaxy')**.

We have previously produced several transgenic 'Galaxy' lines with a wide variation of copy numbers (one through 4) of the *attacin E *gene. Expression of the transgenes in the vegetative tissues was verified by RT-PCR, and the presence of the attacin E protein was determined by western analysis of leaf material. All lines showed expression of the transgenes [21,25], and were diploid, like the non-transformed 'Galaxy'. Several of these lines were rooted and transferred to orchard conditions. The first plants flowered within two to three years after acclimatization in the orchard and continued to produce flowers in the following years. Five transformed 'Galaxy' lines harboring the *attacin E *gene under the control of the PIN2 wound inducible promoter (TGx-157, TGx-158, TGx-161, TGx-164 and TGx-168, all harboring one copy of the transgene, except line TGx-157 and TGx-168, which carry two copies) and two lines harboring the *attacin E *gene under the constitutive promoter CaMV35 S (TGx-165 and TGx-178, both carrying one copy of the *attacin E *gene), as well as the non-transformed 'Galaxy' control plants were successfully pollinated and fruited. Transgenic lines showed no obvious differences in morphology, growth habit, leaf shape (Figure 1A), flower morphology and color, or fruit shape and size (Figure 1B) in comparison to control plants and control fruit. Transgenic and non-transgenic control ('Galaxy') trees and fruit could not be distinguished visually at any time during the growing season or after fruit harvest.

### Impact of the *attacin E *gene on fruit characteristics over time

**Figure 2:**
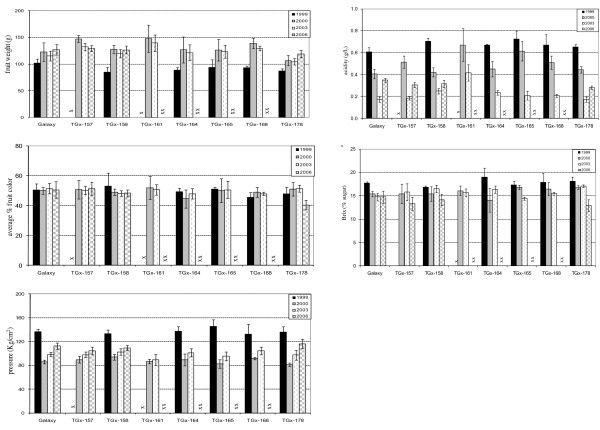
**Comparison of the fruit characteristics (fresh weight, color, firmness, acidity, sugar content) of transgenic fruit compared to non-transgenic fruit (Galaxy) over a period of seven years**. Fruit firmness was determined by a penetrometer and is reported in units of Kg/cm^2^. The acidity index is g/L. Sugar content was measured as degrees Brix or the fraction of sugar per hundred parts aqueous solution, by mass. Stars (x) represent cases where measurements were not taken, in 1999, due to a low number of available fruit and in 2006 (xx), due to the elimination of the trees from the orchard.

Tissues of mature apple fruit from the *attacin E *transformed lines as well as from non-transformed control fruit were analyzed for their fruit characteristics during a period of eight years. Twenty to thirty fruits of transgenic lines coming from two to three different trees were analyzed for their firmness, weight, color, average of acidity and sugar (Figure 2). Fruit characteristics were analyzed in 1999, 2000, 2003 and 2006. TGx-157 and TGx-161 were not included in 1999 evaluation because of insufficient number of fruit. TGx-157, TGx-158, and TGx-178 were also analyzed in 2006, other transgenic lines were not included because the tree had been removed from the orchard. The level of acidity, sugar content (brix), firmness (pressure), fruit weight and color of transgenic fruit could not be distinguished from that of control fruit within any test year. Color and sugar level were very similar between years, however the weight, firmness and acidity level of the fruit varied between years. Yearly variation in fruit firmness and acidity were probably due to yearly variation in degree of fruit maturity at time of harvest. In the first year of production, fruit size was smaller, which is not unusual. The presence of attacin E in the fruits did not have a measurable impact on the five fruit characteristics analyzed compared to the control over the eight year period of fruit production (Figure 2).

### Inheritance of the transgenes

In 2000 and 2006, transformed and non-transformed 'Galaxy' lines were pollinated with 'Idared' pollen. Seeds from transgenic and control 'Galaxy' were harvested, germinated, grown in a greenhouse and evaluated for segregation of the *attacin E *gene by PCR analysis of leaf tissue. The *attacin E *gene segregated 1:1 in the seedling progeny of TGx-158, TGx-161, TGx-164, TGx-165 and TGx-178 (Table 1), the predicted Mendelian ratio for a heterozygote transgenic (T0) crossed with homozygous non-transgenic, and consistent with the integration of one transgene insertion. TGx-157 and TGx-168 segregated with a ratio 2:1 or 1.5:1 (non-transgenic: transgenic), inconsistent with 3 transgenics: 1 non-transgenic ratio expected for two independent T-DNA integrations at different non-linked positions. These segregations were confirmed in 2006 for three transformed lines TGx-157, TGx-158 and TGx-178. The segregation results were also consistent with predicted number of T-DNA inserts obtained from Southern analysis (data not shown).

**Table 1:** Segregation of the *attacin E *gene in 7 independent transformed apple lines determined by PCR analysis of progeny and number of T-DNA insertions determined by Southern analysis.

Lines	2000			2006			Southern Copy number
	Att negative	Att positive	ratio	Att negative	Att positive	ratio	
TGx-157	17	10	1.7:1	46	30	1.5:1	2
TGx-158	13	15	1:1	51	49	1:1	1
TGx-161	21	19	1:1	-	-	-	1
TGx-164	12	11	1:1	-	-	-	1
TGx-165	7	6	1:1	-	-	-	1
TGx-168	25	12	2:1	-	-	-	2
**T**Gx-178	21	24	1:1	42	33	1:1	1
'Galaxy'	35	0		75	0		0

### Presence and distribution of attacin E protein in fruit tissue

To investigate if attacin E was present in fruit tissue, in 2000 we quantified by western blot the amount of attacin E present in the skin tissue of ripe fruit from all the transgenic lines and control. Attacin E was found in fruit tissue of all transgenic lines evaluated (Table 2). To investigate the long-term stability of expression of the *attacin E *gene in fruit of over time, three lines TGx-158, TGx-157 and TGx-178, were evaluated again in 2006 and attacin E protein was present in all of the lines evaluated at similar levels as in the 2000 determination (Table 2).

**Table 2:** Quantification of the amount of attacin E detected in ripe fruit skin samples of different transformed lines expressing the *attacin E *gene, and from the non-transformed control 'Galaxy' in 2000 (for all lines) and 2006 (for TGx-157, TGx-158, and TGx-178).

Lines	pg Attacin/μg of soluble protein	
	**2000**	**2006**

'Galaxy'	0	0

TGx-157	3.71 ± 0.23	3.51 ± 0.25

TGx-158	2.13 ± 0.12	2.76 ± 0.11

TGx-161	4.64 ± 0.36	n.m

TGx-164	4.30 ± 0.48	n.m

TGx-165	3.07 ± 0.12	n.m

TGx-168	4.78 ± 0.14	n.m

TGx-178	3.14 ± 0.16	3.35 ± 0.09

n.m: not measured

**Figure 3:**
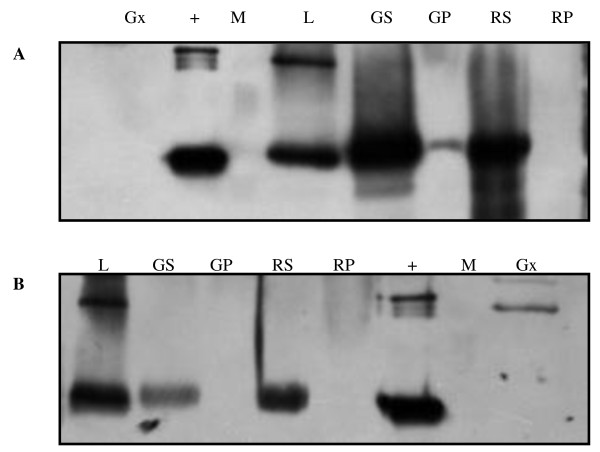
**Western blot analysis of attacin E protein in various tissues: L leaf, GS unripe fruit skin, GP unripe fruit flesh, RS ripe fruit skin, and RP ripe fruit flesh of transgenic TGx-158 (A) and TGx-178 (B), Gx the ripe fruit of non-transformed 'Galaxy' (skin and flesh mixed together), hemolymph containing attacin E (+) and molecular marker (M)**.

Although attacin E protein was found in all the transgenic lines, its distribution in different fruit tissues varied during the season. In 2000, a high expression of attacin E was found in the leaf and in the green or red skin. In contrast none, or a low amount of protein could be detected in the flesh of green fruit and none in the flesh of ripe fruit (red) (Figure 3). Similar results were found in 2006 on the same transgenic lines tested (table 3 and, data not shown).

**Table 3:** Effect of *attacin E *gene on resistance to fire blight over a five year period.

Lines	1999		2001		2002		2003	
	**% shoot infection**	**ranking**	**% shoot infection**	**ranking**	**% shoot infection**	**ranking**	**% shoot infection**	**ranking**

Galaxy	72		39		31		42	

TGx-157	34	2	16.6	1	15.5	3	20.7	2

TGx-158	36	3	28.1	7	18.5	4	29.6	5

TGx-161	51	5	31	6	20	6	30.2	6

TGx-164	70	6	26.8	4	24.4	7	25.1	4

TGx-165	75	7	27.8	5	19.6	5	34.1	7

TGx-168	47	4	24.8	3	15.1	2	22	3

TGx-178	14	1	21.6	2	14.6	1	16.3	1

Vigorously growing shoots were inoculated with *E. amylovora *and the percent of the current season's shoot length that became necrotic determined (% shoot infection).

**Figure 4:**
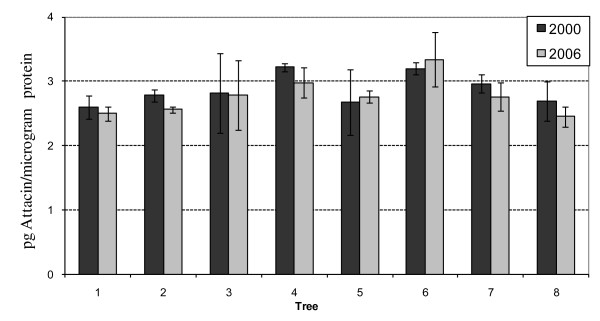
**Quantification of attacin E protein in eight different trees of the transgenic line TGx-158 in 2000 and 2006**. Similar amounts of protein were detected in two independent fruit samples from each tree.

The amount of attacin in the fruit was almost identical in all the transgenic lines, whatever the promoter controlling the expression of this gene. Indeed, no difference in theamount of attacin was observed between the transgenic lines where the attacin gene was controlled by the CaMV35 S promoter (TGx-165 or TGx-178) or by the pin2 promoter (TGx-158, TGx-157) (Table 2). The amount of attacin E was also analyzed in different ripe fruits (skin and flesh) from the same or different trees of lines TGx-158 and TGx-178. The data showed that in 2000, a similar amount of attacin E was found in different fruits from the same or different trees, showing a stability of expression between clonally propagated transformed lines (Figure 4, TGx-178 data not shown). In 2006, this analysis was repeated and similar results were found. The amount of attacin E present in the fruits harvested from the same trees in 2006 showed an amount of attacin E similar to what was found 6 years before, indicating a stability of expression of attacin E during this time (Figure 4).

### Long-term effect of the *attacin E *gene on the resistance to fire blight

In order to characterize precisely the behavior of the transformed lines and the stability of the effect of the *attacin E *gene on fire blight infection, seven transformed lines were acclimatized and planted in the orchard. The trees were inoculated every year, between 1999 and 2003, with *E. amylovora *(Ea 273 10^7 ^cfu ml^-1^). Among the seven transgenic lines expressing the *attacin E *gene, most showed significantly less fire blight infection compared to the control. Over time, the different lines showed a similar response to *E. amylovora *(Table 3) with some difference of ranking (based of the level of resistance) between lines depending on the weather conditions during the year of inoculation. However, over four years of testing for fire blight resistance, the effect of the *attacin E *gene on the susceptibility to fire blight was generally similar and seemed to be stable.

The difference of susceptibility of the control 'Galaxy' between 1999 and the 3 other years is due to the favorable weather conditions for the development of the disease in 1999 compared to the weather in 2001, 2002 and 2003.

## Discussion

Transgenic fruit trees, and apple in particular, have already been obtained in several laboratories [for review [31,32]]. In most cases, transformation and regeneration are not routine, and generally limited to a few examples with relatively low numbers of transgenic lines [33-35]. Genetic engineering offers a possibility to improve woody plants, since it allows the introduction of new desired characteristics into selected superior genotypes. However, the future use of transformed trees on a commercial basis will depend upon thorough evaluation of the potential risk of the modified plants and transgene stability during a prolonged period of time. In this paper we have addressed the stability of the expression of the *attacin E *gene in the orchard during a 12-year period.

Several studies on transgenic trees have found that the transgenic traits can be less stable than originally thought [35]. The expression of the transgenes can be silenced either at the transcriptional or the post transcriptional level. However, recent studies have shown a high stability of the transgenes expression in trees during a short period of time (3 to 4 years) in plant cultivated *in vitro*, the greenhouse or in the field [37,38]. Li et al. [37] observed a very high stability of the GFP and BAR expression in 2256 transgenic poplar tree, derived from 404 primary events, grown over three years in the greenhouse and in the field. Their results suggest that instability of transgene expression in transgenic poplar is low. Flachowsky et al. [38] also found stability of *nptII *and attacin E gene expression in apple cultivated during four years *in vitro*. However, in some of their transgenic lines, the transgene and expression of these genes were unstable; differences were also found between replicate plants of single transgenic lines. Individual plants with partially or completed silenced transgenes were identified as well as plants with no detectable T-DNA. Flachowsky et al. proposed that this instability could be explained by either chimerization or silencing. In support of this hypothesis, a decrease in transgene expression has been associated with the number of *in vitro *subcultures [39].

In apple transgene expression was previously monitored using histochemical GUS staining over a 4-year period in transgenic Royal Gala lines in greenhouse [16]. GUS expression was detected in leaf tissues of transgenic trees in each growing season, and in the reproductive organs of transgenic trees that produced flowers and fruit. These data confirm the results of James et al. [2,15], who showed stable expression of two transgenes, nopaline synthase and *npt*II, in transgenic 'Greensleeves' apple, using enzyme extraction methods. In our study we confirmed these two previous reports by monitoring the expression level of the *attacin E *gene in different parts of the tree, under orchard conditions, over 12 years. Stable expression of the *attacin E *gene was observed in both leaves and fruit and in different trees of the same clone. Indeed, the level of expression was very similar in different fruit from the same tree or from different trees of the same clone.

The *attacin E *gene was expressed more in the skin than in the flesh. However, the expression of the *attacin E *gene in the fruit did not affect fruit shape and size, acidity, firmness, or sugar level, or tree morphology, leaf shape, flower morphology, or color. These data were confirmed several times where the transgenic lines were grown under orchard conditions. Ruhmann et al. [40] also showed that expression of a stilbene synthase gene does not affect the leaf shape, flower morphology or color, or fruit shape and size compared to control plants and fruit of *Malus x domestica *(cv. Elstar' and 'Holsteiner Cox) Dandekar et al. [34] and Hrazdina et al. [41] produced transgenic apple fruit modified in their capacity to synthesize endogenous ethylene. Ethylene suppressed fruits were significantly firmer than controls and displayed an increased shelf-life, but no significant difference was observed in sugar or acid accumulation suggesting that sugar and acid composition were not altered, consistent with our data. However, a significant and dramatic suppression of the synthesis of volatile esters was observed due to the inhibition of ethylene [42-44].

The *attacin E *transgenic trees produced normal seeds, which enabled us to test the inheritance of transgene activity in progenies. No difference in seed production between the transgenic and the non-transgenic lines was observed (data not shown). Using these seeds, the presence of the *attacin E *gene was evaluated by PCR for seven transgenic lines in 1999 and three transgenic lines in 2006. In the progeny of five lines that had a single copy of attacin E according to Southern analysis, a 1:1 segregation ratio for the presence of *attacin E *was observed. This expected Mendelian segregation persisted in the same trees in trials separated by six years. In two other transgenic lines, Tgx-157 and Tx-168, for which Southern blot data indicated two copies of attacin E, variable segregation was observed, not fitting any expected Mendelian segregation. The reasons for this non-Mendelian behavior in the inheritance of attacin E when it was present as two copies is not clear. However for breeding purposes, it is evident that transgenics with a single copy of the desired transgene should be used

The stability of expression of *attacin E *over a period of 12 years in the orchard is supported by the fact that the resistance to fire blight of these different transgenic lines was conserved at similar level during the several years of the trial. This duration of phenotype stability for resistance provided by a transgene in a tree fruit has not been previously reported.

## Conclusions

We demonstrate the stable integration and expression of a transgene (*attacin E*) in apple for more than 12 years under orchard conditions. Expression of this gene resulted in an increase in resistance to fire blight throughout these years and had no effect on tree morphology, fruit morphology or internal fruit quality characteristics. This report shows that it is possible to improve the resistance of apple cultivars through genetic engineering, without adverse modification of fruit characteristics during long term cultivation.

## Materials and methods

### Plant Material

'Galaxy' apple (*Malus Xdomestica *Borkh.) plants harboring the *attacin E *gene under the control of an inducible (wound inducible proteinase inhibitor II promoter from potato; has a low level of constitutive expression in apple) or constitutive promoter (CaMV35S) were previously produced [21]. Transgenic 'Galaxy' apple trees were produced in 1994 and planted in the field in 1995. Fruits were obtained from several transgenic lines (events) grown in a research orchard in Geneva, New York, from 1999 through 2006.

### Determination of ploidy level

Ploidy level in the transgenic and non-transformed lines was estimated by flow cytometry. Nuclei were isolated from *in vitro *leaves by manual chopping with a razor blade directly into the buffer described by the manufacturer (Partec, Münster, Germany). After addition of 4,6 diamino 2 phenyl indole dihydrochloride (DAPI; 2% v/v) and filtration through a 20 μm nylon mesh, the mixture was analyzed with a flow cytometer (Partec II; Partec).

### Determination of fire blight resistance

The level of resistance of transgenic lines was evaluated in the field for several years (1999 to 2004). Actively growing shoots were inoculated by cutting the youngest expanded leaf with scissors previously dipped in an *E. amylovora *suspension (strain Ea273, 10^7 ^cfu ml^-1^). Disease symptoms were rated at 2 months after inoculation by measuring the length of the necrosis and the total length of the current season's shoot growth. Inoculated shoot tips became orange-brown and eventually dark brown, often with production of cloudy ooze dropets which darkened with tim. These symptoms proceeded basipetally on the inoculated shoot, and eventually terminated depending on the susceptibility of the shoot resulting from its genetics and the vigor if its growth. After the necrotic lesion ceased extension, an axillary bud just below the lesion margin broke and proceeded to grow. Inoculated hoots with no symptoms two months after inoculation but which stopped growing during this period were discarded from the analysis.

### Determination of attacin protein concentration

Attacin content in plant tissue was determined as described by Ko et al. 1999 [25]. Protein extraction in Bradley buffer was carried out from leaves, and skin and flesh of fruit at two stages, green and red, from each transgenic line in the field. Protein extracts were quantified against a bovine serum albumine (BSA) standard using a colorimetric assay according to Bradford [26]. For western analysis, 10 μg aliquots of protein extract from control and transgenic clones in Laemmli buffer were separated on 4-20% Tris Glycine polyacrylamide gel (PAGEr^® ^Gold Precast Gel, Biorad) according to the manufacturer's instructions. After electrophoresis, proteins were blotted onto immobilon-P PVDF membrane (Millipore) by active transfer. To confirm that equal amounts of protein were loaded for all samples, the membranes were stained with Ponceau S after transfer (data not show). Strain was washed from the membrane prior to blocking. After blocking, membranes were incubated at 4°C overnight in attacin antibodies (diluted at 1/10,000) with gentle agitation. The membranes were rinsed 4 times for 5 min in PBST and then incubated at room temperature for 2 h in donkey anti-rabbit (Amersham Life Science, Buckinghamshire, UK), conjugated to horseradish peroxidase. Membranes were rinsed as above. Protein bands were detected on Classic CX Xray film (Laboratory Products Sales, Rochester, NY) using a chemiluminescence substrate (SuperSignal, Pierce, Rockford, IL).

Protein bands detected on immunoblot film were digitized to an electronic image, and the intensity of the band image was measured using Image 1.59 software (National Institutes of Health, Bethesda, MD). To quantify [ng (mg apple leaf)^-1^] the amount of attacin in transgenic plants, hemolymph of immunized *H. cecropia *containing attacin (12.5, 25, 50, 100, and 200 units) was loaded onto one SDS-PAGE gel. A 200 unit standard was included on each additional gel to adjust for membrane variability. The intensity of each sample and standard was adjusted by subtracting background intensity from the measured intensity. A standard curve between the ln ('1 unit') of hemolymph and the measured image intensity of the standard was calculated using CA-Cricket Graph III Version 1.5.2 (Computer Associates International Inc., Islandia, NY). The ratio (intensity of 200 units on standard membrane per intensity of 200 units on sample membrane) was calculated, the intensity of a sample was multiplied by the ratio, and the adjusted intensity value was used to estimate attacin concentration from the linear regression equation derived from the standards.

### Southern and PCR analysis

DNA was extracted from the leaf tissue of non-transformed and putative transgenic plants [27]. Genomic DNA was digested with *HindIII *or *EcoRI*, electrophoretically separated on 0.8% agarose gel, and transferred to a nylon membrane Genescreen Plus as described by the manufacturer (NEN Research Products, Boston). Probes were the nptII (750 bp) and attacin coding regions labeled with 32^P ^using random primers [28]. PCR analysis for the presence of the attacin gene in seed was conducted as described by Ko et al. [21,29].

### Fruit characteristics

Fruit characteristics were determined by measuring parameters including acidity, sugar (Brix), firmness, weight and color. A 20-30 fruit sample of transgenic lines and non-transformed control were collected, and graded by a commercial computerized fruit weight and color sorter (Pomone MAF Roda, Traver, CA). On the same fruits the firmness, sugar and acidity content were determined. Fruit firmness (kg·cm^-2^) was assessed using an electronic firmness tester (model EPT-II, Lake City Technical Product Inc., Kelowna, BC, Canada) fitted with an 11-mm diameter tip. Acidity (%), and total sugars (Brix) were analyzed according to the method of the Official Methods of Analysis [30].

## Competing interests

The authors declare that they have no competing interests.

J.L. Norelli and H.S. Aldwinckle are inventors on US patents 6,100,453 and 5,824,861 "Transgenic pomaceous fruit with fire blight resistance", but neither patent has been licensed.

## Authors' contributions

EBW carried out the western blot analysis and fruit analysis. JLN co-conceived the study, initiated the experiment, participated in the experimental design, carried out some of the plant inoculation and contributed to writing of the manuscript and its revision. HSA Co-conceived the study and contributed to the manuscript revision. MM participated in the experimental design, carried out the molecular analysis, some pathogenicity test, and wrote the first manuscript draft, and its revision. All authors read and approved the final manuscript.
